# Refined 3D Urban Building Reconstruction from TomoSAR Point Clouds via Multi-Level Geometric Priors and Shadow Analysis

**DOI:** 10.3390/s26134028

**Published:** 2026-06-25

**Authors:** Wenkang Liu, Haoyuan Chen, Jinsong Zhang, Cheng Qian, Gang Xu, Ning Li, Guangcai Sun, Mengdao Xing

**Affiliations:** 1 The School of Information Mechanics and Sensing Engineering, Xidian University, Xi’an 710071, China; zhangjinsong@xidian.edu.cn; 2 The Guangzhou Institute of Technology, Xidian University, Xi’an 710071, China; 23021211943@stu.xidian.edu.cn; 3 Shanghai Aerospace Electronic Technology Institute, Shanghai 201109, China; chenquancc@163.com; 4 The State Key Laboratory of Millimeter Waves, Southeast University, Nanjing 210096, China; gangxu@seu.edu.cn; 5 The Faculty of Infor-X, The School of Information Mechanics and Sensing Engineering, Xidian University, Xi’an 710071, China; xmd@xidian.edu.cn; 6 The National Key Laboratory of Radar Signal Processing, Xidian University, Xi’an 710071, China; gcsun@xidian.edu.cn

**Keywords:** SAR tomography (TomoSAR), point cloud, 3D model reconstruction, regularization

## Abstract

Reconstructing building models from urban SAR tomography (TomoSAR) point clouds is often constrained by limited resolution, low positioning accuracy in elevation, as well as data incompleteness and artifacts caused by microwave imaging mechanisms. These challenges seriously restrict the extraction of high-accuracy building models with structural details from TomoSAR point clouds. This paper proposes a refined urban building modeling method that effectively utilizes structural priors, including directionality, orthogonality, and potential symmetry. First, a piecewise fitting strategy integrated with density-based segmentation is employed to iteratively estimate the main directions of the buildings and capture finer geometric variations of complex façade footprints than simple-plane approximations. Second, a roof extraction algorithm combining an adaptive Doug-las–Peucker approach with symmetry evaluation and constraints is developed to regularize roof outlines and repair data defects. Crucially, to handle extreme cases where roof data are entirely missing, a novel building width estimation method based on building shadow analysis is proposed. Experiments conducted on the SARMV3D-1.0 and SARMV3D-3.0 point cloud datasets demonstrate that the proposed method significantly enhances reconstruction accuracy and geometric fidelity in urban regions compared to state-of-the-art approaches.

## 1. Introduction

Synthetic aperture radar (SAR), as an all-day and all-weather earth observation sensor, can achieve wide-range, full-time information acquisition on the earth surface, including rich building structural information in urban regions. Therefore, it can be used to construct three-dimensional building models, which have significant application potential in urban emergency response and disaster management [1], urban planning [2], telecommunications network planning [3], and the construction of urban digital twins [4,5]. Many studies have attempted to detect and identify building features from two-dimensional SAR amplitude images and complete rough model extraction [6,7,8,9]. However, urban scenes are highly complex, and SAR images are affected by inherent imaging defects such as coherent speckle noise and layover [10], making it significantly challenging to directly extract high-precision building features from 2D SAR images [11]. SAR tomography (TomoSAR), as an advanced radar imaging technology, aims to achieve true three-dimensional [12,13,14,15] or four-dimensional synthetic aperture radar imaging [16,17,18]. It can separate multiple scatterers within a single resolution cell while simultaneously inverting their elevation information [19]. High-quality TomoSAR point clouds can be produced for reconstructing large-scale urban building models [20,21,22].

However, due to the complexity of urban environments and the inherent limitations of microwave imaging, the TomoSAR-derived 3D point clouds exhibit missing data segments, limited geolocation accuracy, and significant multipath artifacts. These issues pose significant challenges for building model reconstruction. TomoSAR point clouds typically exhibits the following three characteristics. First, due to the constrained observation geometry of SAR tomography, only building surfaces within the radar illumination sector generate detectable scatterers, resulting in persistent data voids on occluded façades that hinder complete 3D reconstruction [23]. Additionally, although roofs are typically fully illuminated, the SAR imaging mechanism results in both point cloud voids and heterogeneous density distributions. Moreover, non-architectural strong scatterers (e.g., water tanks, solar panels) on roofs become aliased with building point clouds, causing geometric discrepancies between the point cloud outlines and actual building shapes. Multipath ghost artifacts represent another prominent issue. Multipath reflections between buildings and the ground create false scatterers, appearing as ghost points around or inside structures [24,25]. These coherent artifacts are hard to remove with simple noise filtering [23,26,27] and complicate building point cloud segmentation in dense urban areas.

To mitigate these inherent imaging limitations, leveraging structural priors is a widely adopted approach to constrain reconstruction results. Such constraints can be applied during SAR tomography, model extraction, or jointly. In the tomography stage, spatial regularization terms have been introduced to constrain point distribution [28]. Additionally, a multi-look generalized likelihood ratio test (GLRT) was proposed for scatterer detection [29], while the local plane model was adopted to handle complex structures like sloped roofs [30]. During model extraction, the verticality of façades was utilized in [31] to derive key parameters. Similarly, weighted alpha shapes were employed in [20] to estimate principal directions for regularizing roof outlines. Furthermore, structural priors can refine tomographic results, such as the Z-shape prior [32] and graph-cut-based surface segmentation [33], which enable iterative updates for precise modeling. Although deep learning (DL) has shown effectiveness in tomographic inversion [27,34,35,36] and 2D footprint extraction [7], and visual reasoning for Earth observation [37], its application to downstream 3D modeling faces significant hurdles. Unlike dense LiDAR data, TomoSAR point clouds are characterized by sparsity, occlusion, and artifacts, which constrain the transfer of existing networks. Moreover, the scarcity of annotated 3D datasets limits robust supervised training.

Incorporating prior knowledge about real-world structures helps constrain large solution spaces caused by insufficient data, enabling more reliable results, while different constraints may produce inconsistent results. For example, simple geometric priors often cannot accurately represent real building structures, reducing model accuracy. Therefore, more refined and realistic structural priors are needed to regularize the reconstruction results. This paper refines the regularization constraints at the point cloud reconstruction model stage to achieve more accurate and reliable 3D reconstructions. The main contributions are summarized as follows:

(1) The proposed method refines regularization constraints for both façade and roof reconstruction by leveraging geometric priors. For façades, it exploits the fact that geometric variations usually align with principal and orthogonal axes. By utilizing statistical analysis of façade segments and orthogonality constraints, the method enhances the reconstruction performance of complex structures. Simultaneously, for roofs, a strategy combining principal-direction regularization with optimal outline fitting is developed, which employs adaptive parameters and integral error minimization to achieve high-fidelity structural regularization.
(2) An optional symmetry constraint is introduced for roof reconstruction. Utilizing the extensive symmetry inherent in architectural structures [38], constraints are applied to roof point clouds with symmetric features following architectural symmetry evaluation. This process filters outlier points and completes building outlines, leading to enhanced regularization capability and excellent performance in building model reconstruction from irregular TomoSAR point clouds.
(3) A building parameter estimation method is proposed for cases of severely missing roof point clouds. Based on a rectangular roof assumption, reliable width estimation is achieved through ground projection area analysis combined with façade parameters, enabling the reconstruction of severely missing roof structures.

## 2. Overview of the Processing Workflow

The method begins with extracting and segmenting point cloud data of different buildings. For each building, the roof and façade point clouds are then separated and undergo regularized reconstruction. The regularized reconstruction process comprises three hierarchical levels: global orientation regularization, detailed outline regularization, and optional symmetric regularization. First, the main orientation and other primary parameters are extracted to determine the overall outline trend. Subsequently, structural priors are employed to constrain local outline details. To further obtain complete outlines, symmetry can be optionally introduced as an additional constraint for outline refinement. The multi-level regularization strategy effectively integrates both macro- and micro-scale geometric structural priors, enabling improved architectural model reconstruction.

**Figure 1:**
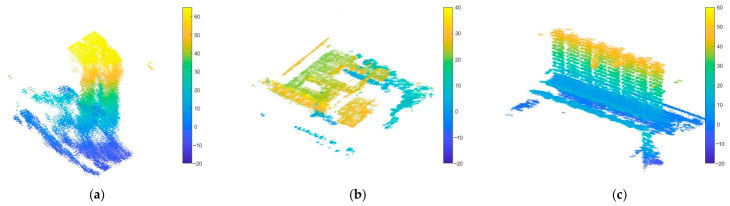
Building point clouds under three different scenarios: (**a**) Case 1: point cloud data containing both façade and roof; (**b**) Case 2: point cloud data containing only roof; (**c**) Case 3: point cloud data containing only façade.

**Figure 2:**
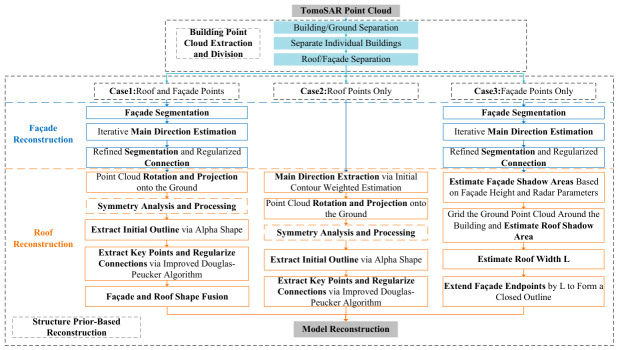
Flowchart of the proposed method.

Figure 1 shows three reconstruction scenarios based on the availability of roof and façade point clouds: datasets with both roof and façade points, roof-only cases, and façade-only cases. The underlying causes are examined in [20]. Figure 2 illustrates the processing workflow for detailed urban building reconstruction from TomoSAR point clouds using structural priors. The processing workflow initiates with building point cloud extraction through a combined simple morphological filter (SMRF) and density-based segmentation approach, followed by the separation of distinct buildings using density clustering. For individual buildings, façade height estimation is performed, followed by separation of roof and façade point clouds, leading to the aforementioned three reconstruction scenarios.

**Case 1**: With both roof and façade point clouds available, structural information extracted from façade footprints, including principal orientation and construction details, can be utilized to reconstruct refined outlines. The principal orientation serves as a fundamental parameter for regularization, for which we developed a progressive segmentation algorithm that simultaneously estimates orientation and reconstructs refined footprints. Extracting building outlines from roof point clouds presents unique challenges due to interference from non-building objects and point cloud gaps caused by SAR coherence issues. To address these challenges, we implement a symmetry-based regularization framework that first evaluates architectural symmetry through symmetry metrics, then corrects asymmetric components in structures that should be symmetric. To extract more regular and reasonable outlines, we improve the Douglas–Peucker algorithm with adaptive threshold selection for key feature points of roof alpha shapes. These points are then connected under area constraints to reconstruct regularized outlines. Finally, integrating roof outlines with refined façade footprints produces the complete building model with enhanced architectural details.

**Case 2**: This scenario typically occurs in low-rise buildings where mutual obstruction between buildings results in only roof point clouds being available. In such cases, the directional information and structural details provided by the façade cannot be utilized, leaving only the roof point cloud for building outline reconstruction. However, we can still estimate the building’s main orientation by analyzing edge line segments of the roof outline using weighted methods. Once the main orientation is determined, the subsequent roof outline extraction process is the same as in Case 1, including symmetry analysis, feature point extraction, and regularized outline generation.

**Case 3**: In certain scenarios, building roofs produce weak radar backscatter signals, leaving only façade point clouds available. In single-perspective TomoSAR point clouds, only façade point clouds from one side are observable, making full outline reconstruction challenging. Previous studies [20] did not consider outline reconstruction under such conditions. To address this challenge, we propose a roof parameter estimation method based on building shadow analysis in sparsely built areas where building shadows can be extracted. Given the linear propagation of radar signals, even when roof data are missing, we can estimate roof parameters by analyzing building shadows to support modeling.

The proposed method enables reconstruction of more refined façade structures when applying symmetry priors to optimize shape outlines, thereby generating more reasonable building outlines. Furthermore, for the challenging Case 3 situation, it presents a feasible reconstruction approach that estimates roof parameters and reconstructs outlines even in the absence of roof data.

Section 3 and Section 4 detail the principles and implementation of the proposed method.

## 3. Main Processing Steps

### 3.1. Building Point Cloud Extraction and Segmentation

To refine each building according to its structural characteristics, individual building point clouds must be isolated while mitigating interference from ground points and multipath artifacts. Since roofs and façades require different regularization approaches, accurate segmentation of building point clouds into roof and façade components is crucial. The completeness and accuracy of this segmentation will directly determine the precision and quality of subsequent 3D reconstruction.

#### 3.1.1. Building Point Cloud Extraction

The methodology begins with the simple morphological filter (SMRF) [39], a standard algorithm for LiDAR ground segmentation. SMRF operates by generating a gridded digital surface model (DSM) and identifying initial ground points via lowest-elevation analysis. It then applies morphological opening operations to progressively filter out non-ground objects based on elevation differences. However, directly transferring this LiDAR-optimized method to TomoSAR point clouds is problematic. TomoSAR data often lead to misclassifications, such as identifying flat rooftops as ground surfaces or mistaking high-elevation multipath artifacts for legitimate building structures.

To address these limitations, we incorporate density and height constraints into the segmentation process. First, we apply a height constraint to correct rooftop misclassification. Since rooftops are structurally elevated, points classified as ground by SMRF but exhibiting significant height relative to the estimated ground plane are re-categorized as building points. Second, we leverage the vertical nature of building façades to filter multipath noise. Because vertical façades produce high-density clusters when projected onto the ground plane—unlike the scattered distribution of multipath artifacts—a projection density analysis allows for the effective separation of true building points from noise, thereby significantly enhancing the segmentation accuracy.

#### 3.1.2. Roof-Façade Segmentation

**Figure 3:**
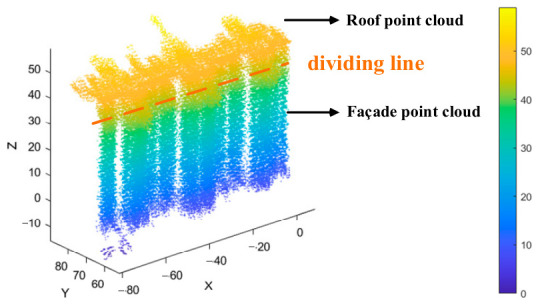
Single-building segmentation results with height-coded point coloring. Orange line marks the segmentation boundary between façade and roof point clouds according to the estimated façade height.

Since building façades are generally perpendicular to the ground, the façade point cloud can be extracted by projecting the point cloud onto the ground plane and analyzing the density distribution [20,31]. However, grid partitioning and density-threshold-based operations often compromise the integrity of the façade point cloud. To address this, the proposed method improves segmentation by incorporating estimated façade height information. Specifically, the façade height is estimated through density analysis of the projected point cloud, and this height is subsequently used as a threshold to separate roof points from façade points. This approach effectively preserves edge details and reduces interference, ensuring more precise segmentation than simple density-based projection, see Figure 3.

### 3.2. Façade Regularization Reconstruction

Leveraging the verticality and orthogonality inherent in urban structures, façade extraction is typically modeled as a line-fitting problem. However, applying a global fit to complex buildings often leads to detail loss, as irregular features may be smoothed out. To address this, we propose an optimized piecewise fitting strategy. This method iteratively refines the principal direction by segmenting the point cloud along the estimated axis, performing local fitting, and then aggregating these local orientations. This approach balances the need for architectural regularity with the preservation of geometric accuracy. The detailed workflow is as follows:

**Step 1:** Project the building point cloud onto the ground plane. Apply a line-fitting algorithm to estimate the initial principal direction, denoted as $\theta_{0}$ relative to the *x*-axis.

**Step 2:** Rotate the point cloud to align with the *x*-axis based on the current angle $\theta_{0}$, and project it onto the *xy*-plane. Divide the projected data into intervals along the *x*-axis with a width of Δx. For each interval, calculate the median y-coordinate, denoted as $med{\left(y\right)}_{i}$, and compute the depth difference $\Delta med{\left(y\right)}_{i}=med{\left(y\right)}_{i}-med{\left(y\right)}_{i+1}$ between adjacent intervals.

**Step 3:** Define a threshold μ to detect structural depth jumps. If the difference satisfies $\left|\Delta med{\left(y\right)}_{i}\right|\;>\;\mu$, the boundary between these intervals is marked as a segmentation point, effectively dividing the façade into distinct clusters.

**Step 4:** Partition the point cloud using the detected segmentation points. Apply the RANSAC algorithm to fit a linear segment to each partition and estimate its specific tilt angle $\theta_{k}$. To ensure robustness, segments with tilt angles that deviate significantly from the global trend $\theta_{0}$ are considered outliers and excluded from the subsequent weighting process.

**Step 5:** Update the global principal direction $\theta_{new}$ by performing a weighted vector averaging of the valid local segments

(1) $$\theta_{new}=\arctan2\left(\sum_{i=1}^{m}w_{i}\sin(\theta_{i}),\sum_{i=1}^{m}w_{i}\cos(\theta_{i})\right)$$

where $\theta_{i}$ represents the tilt angle of the ith valid segment, and $w_{i}$ is the normalized weight proportional to the number of points within that segment.

**Step 6:** If the update magnitude $|\theta_{new}-\theta |<\epsilon$ (where ϵ is the tolerable error), output $\theta_{new}$ as the final principal direction. Otherwise, set $\theta_{0}=\theta_{new}$ and return to Step 2 for the next iteration.

**Figure 4:**
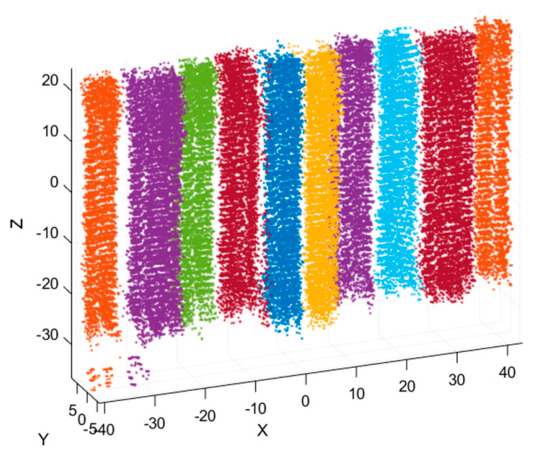
Initial segmentation results of a single façade (color-coded by segment with point clouds aligned parallel to the *x*-axis).

**Figure 5:**
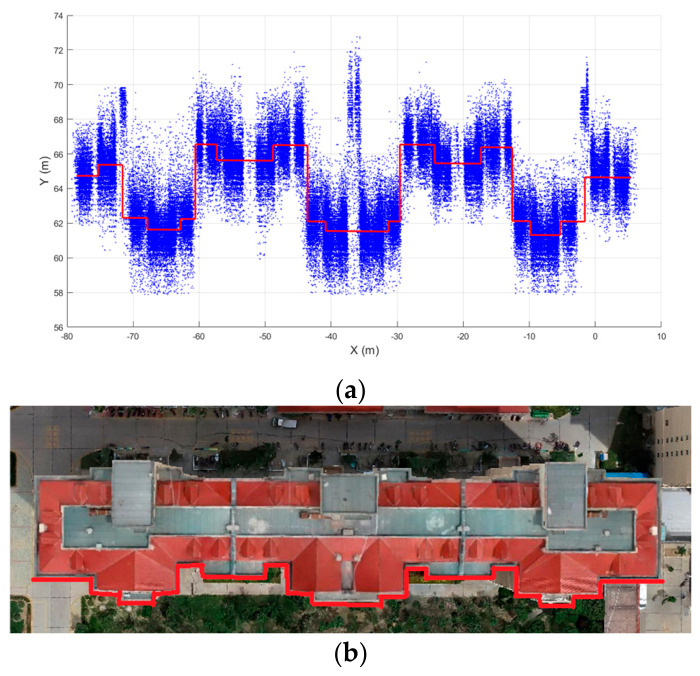
Final façade footprint reconstruction and comparison. (**a**) Horizontally and vertically connected footprint outline. Blue points show building point cloud (top view), red lines indicate fitted footprint results. (**b**) Real façade outline.

As shown in Figure 4, the point cloud is first rotated to align with the principal direction for preliminary segmentation. To capture finer geometric variations, the data are divided into fine spatial intervals to calculate median coordinates, which are smoothed via a moving average to filter outliers. Segmentation is then performed based on median differences and spacing constraints. Finally, these segments are locally fitted and connected perpendicularly to construct a continuous, orthogonal façade footprint, as illustrated in Figure 5a.

### 3.3. Regularized Roof Reconstruction

While refined façade footprints contribute essential structural details, they are often incomplete due to radar occlusion. Consequently, roof point clouds become indispensable for recovering the complete building morphology. However, directly extracting accurate outlines from these data is challenging; the low positional accuracy of TomoSAR point clouds—coupled with interference points and noise—results in highly irregular edge distributions that deviate from the actual building structure.

#### 3.3.1. Principal Direction Estimation in the Absence of Façades

In scenarios where façade point clouds are sparse or absent (e.g., low-rise buildings), the principal orientation must be inferred solely from the roof geometry. We propose a robust estimation method based on weighted orthogonal histogram analysis of the roof footprints.

The alpha shape algorithm [40] is first employed to extract the boundary polygon from the discrete roof point cloud. The boundary consists of a set of line segments $\left\{s_{1},s_{2},\ldots ,s_{m}\right\}$. We calculate the length $L_{i}$ and orientation angle $\phi_{i}\in [0,\;\pi )$ for each boundary segment. The angular domain is discretized into K bins of width Δϕ. A raw directional histogram H(⋅) is constructed by accumulating the lengths of edges falling into each bin

(2) $$H(\theta_{k})=\sum_{i=1}^{n}L_{i}\cdot I(\phi_{i}\in {\mathrm{bin}}_{k})$$

where I(⋅) is the indicator function.

To leverage the inherent orthogonality of urban structures, we enforce a constraint where edge segments separated by $90^{\circ }$ contribute to the same principal axis estimation. We define a composite score $S(\theta_{k})$ that aggregates contributions from orthogonal directions

(3) $$S(\theta_{k})=H(\theta_{k})+H(\theta_{k}+\frac{\pi }{2}),\;\forall \theta_{k}\in [0,\;\frac{\pi }{2})$$

The principal direction $\theta_{main}$ is determined by identifying the peak of this composite score:

(4) $$\theta_{main}=\arg\max_{\theta_{k}}S(\theta_{k})$$

**Figure 6:**
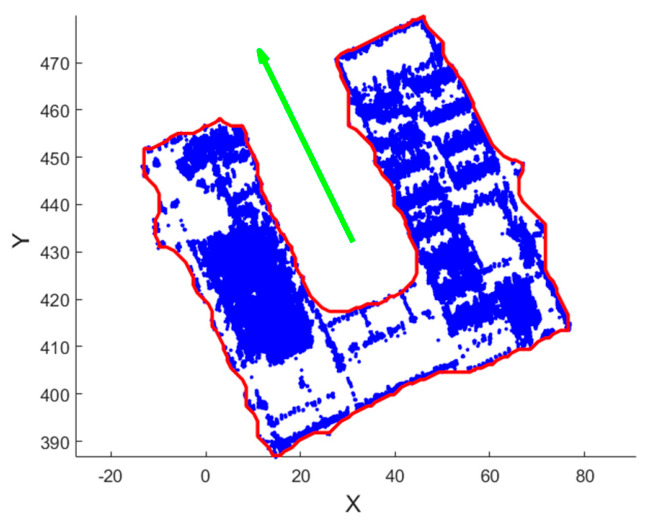
Schematic diagram of principal direction estimation from building point cloud without façade. The blue point cloud shows a top view of the roof point cloud, the red polyline represents the alpha shape extraction result, and the green line segment indicates the estimated building principal direction.

This strategy ensures that the estimation is supported by the global geometric distribution, making it robust against local boundary fragmentation or noise. Figure 6 illustrates the estimated principal orientation of a building using the proposed method.

#### 3.3.2. Refined Roof Outline Extraction Based on Alpha Shape and Douglas–Peucker Algorithm

Direct application of the alpha shape algorithm to TomoSAR data typically yields noisy, jagged boundaries due to the sparsity of roof points. To construct regularized building footprints, we propose an enhanced simplification framework based on the Douglas–Peucker (DP) algorithm, incorporating adaptive thresholding and orthogonality constraints. The Douglas–Peucker algorithm is a curve simplification technique that reduces data complexity by decreasing the number of points representing a curve whilst preserving its overall geometric characteristics [41,42].

**Figure 7:**
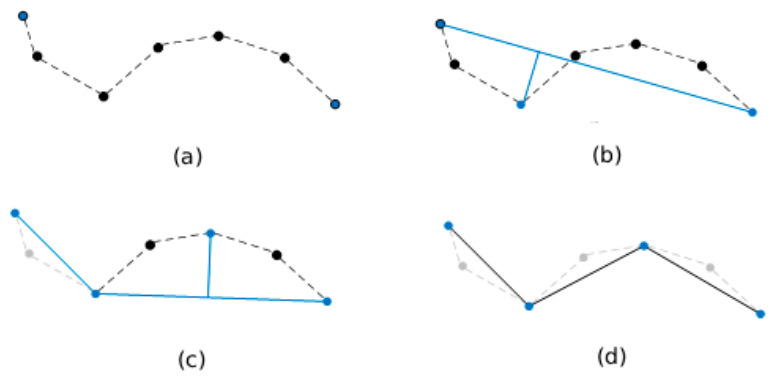
Schematic diagram of the Douglas-Peucker algorithm. (**a**) The original curve represented by a set of points (black dashed line). (**b**) Finding the point with the maximum perpendicular distance to the baseline connecting the start and end points. (**c**) Splitting the curve at the key feature point and recursively repeating the process for the newly formed segments. (**d**) The final simplified polyline. In the figure, the black dashed lines represent the original point sequence, the blue solid lines represent the simplified segments, and the blue dots indicate the retained key feature points.

As illustrated in Figure 7, the algorithm operates recursively as follows: It initializes by establishing the curve’s starting point $p_{start}$ and ending point $p_{end}$ as the baseline segment. For each intermediate point $p_{i}$, the algorithm calculates its perpendicular distance to the line segment $\bar{p_{start}p_{end}}$ and identifies the point $p_{m}$ exhibiting the maximum distance $d_{m}$. If $d_{m}$ exceeds a predefined threshold ψ (which determines the degree of simplification), $p_{m}$ is retained as a key feature point. The curve is subsequently divided at $p_{m}$ into two sub-segments, and the same process is applied recursively to each segment. This iteration continues until all points fall within the threshold, resulting in a set of key feature points that effectively represent the essential geometric characteristics of the original curve.

**Figure 8:**
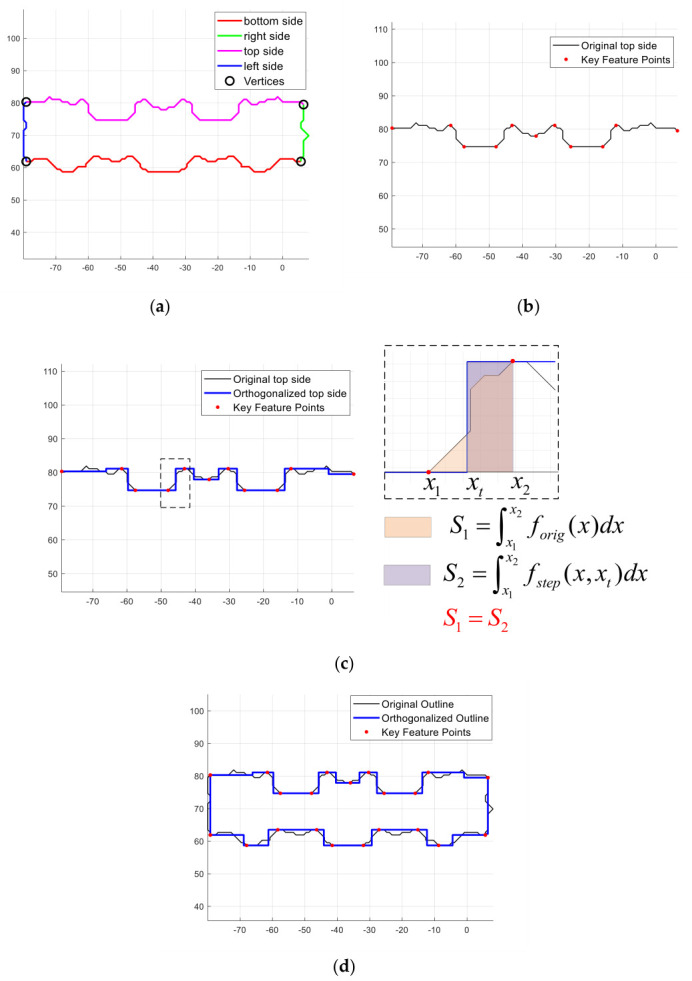
Schematic diagram of orthogonal regularization using the Douglas–Peucker algorithm. (**a**) Four edges with different orientations divided based on extremum point analysis. (**b**) Taking the top edge as an example, key feature points detected by the Douglas–Peucker algorithm. (**c**) Determination of turning points between two key feature points: orange and purple scribbles represent the original outline integral and orthogonal outline integral between two feature points, respectively. When $S_{1}=S_{2}$ is satisfied, $x_{t}$ is determined as the optimal turning point. (**d**) Final orthogonalized outline.

First, the raw alpha shape polygon is analyzed to identify dominant geometric corners (extrema points in the local coordinate system). These corners serve as anchor points to divide the closed polygon into a set of distinct edge segments (see Figure 8a). This segmentation allows for piece-wise processing, which is robust to complex shapes (e.g., U-shaped or L-shaped structures) where a single global fit is inappropriate.

Standard DP algorithms rely on a fixed distance threshold ψ, which often leads to either over-simplification (loss of detail) or under-simplification (retention of noise). We propose an adaptive threshold optimization method. For each segment, we iterate through a candidate set of thresholds $\left\{\psi_{1},\psi_{2},\ldots ,\psi_{m}\right\}$ and select the optimal $\psi^{*}$ that maximizes a composite quality metric M:

(5) $$M(\psi )=w_{p}\cdot \mathrm{IoU}\left(P_{\mathrm{orig}\;},P_{\psi }\right)+w_{r}\cdot R\left(V_{\mathrm{orig}\;},V_{\psi }\right)$$

where $P_{\mathrm{orig}\;}$ and $P_{\psi }$ denote the polygon areas formed by the original and simplified outlines, respectively; $\mathrm{IoU}\left(\cdot \right)$ represents the intersection-over-union (Jaccard Index) of the areas, measuring geometric fidelity; and $R\left(\cdot \right)$ is the vertex reduction rate, defined as

(6) $$R=1-\frac{\left|V_{\psi \;}\right|}{\left|V_{\mathrm{orig}\;}\right|}$$

where $\left|V\right|$ is the vertex count. The weights $w_{p}$ and $w_{r}$ balance the trade-off between shape preservation and simplification efficiency.

The simplified key points are connected using an orthogonal constraint to enforce architectural regularity. For any two adjacent key points $p_{1}\left(x_{1},\;y_{1}\right)$ and $p_{2}\left(x_{2},\;y_{2}\right)$, the optimal turning point $x_{best}$ for the orthogonal connection is determined by minimizing the integral area difference between the original curve $f_{orig}\left(x\right)$ and the stepped path $f_{step}\left(x,\;x_{t}\right)$ (as schematically demonstrated in Figure 8c):

(7) $$x_{best}=\underset{x\in [x_{1},x_{2}]}{\arg\min}\left|\int_{x_{1}}^{x_{2}}f_{orig}(x)dx-\int_{x_{1}}^{x_{2}}f_{step}(x,x_{t})dx\right|$$

**Figure 9:**
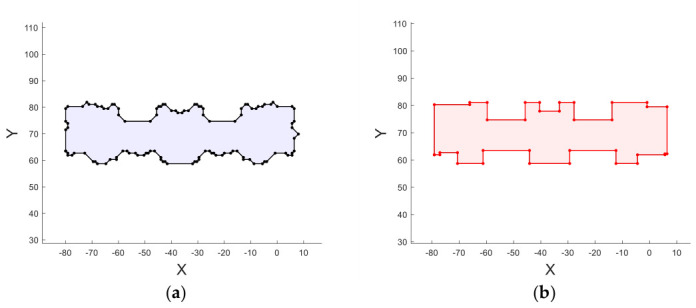
Comparison of roof outline regularization results. (**a**) Original outline extracted via the alpha shape algorithm. (**b**) Regularized outline after orthogonalization using the adaptively selected threshold.

This integration ensures that the reconstructed orthogonal footprint (shown in Figure 8d) preserves the area distribution of the original point cloud data, see Figure 9.

### 3.4. Fusion of Façade and Roof Outlines

**Figure 10:**
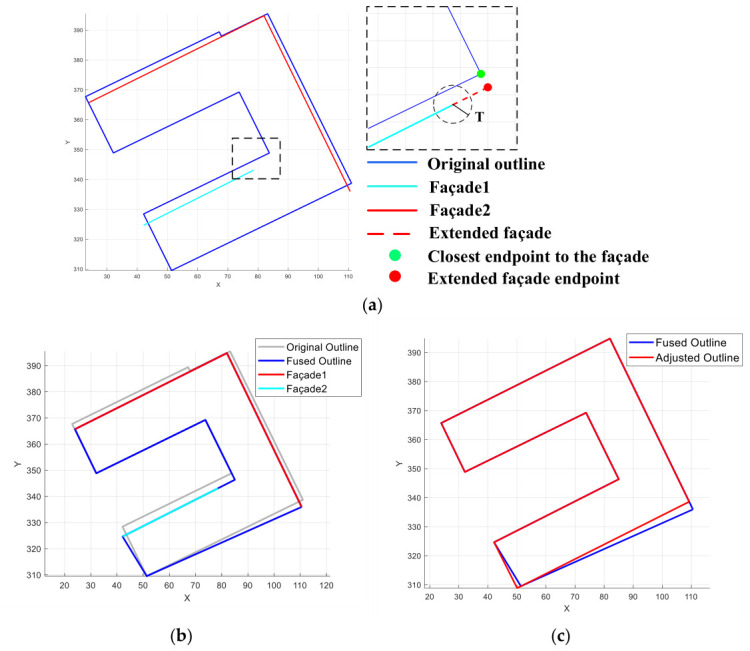
Schematic diagram of the façade–roof fusion mechanism. (**a**) Linear extension strategy: When a roof vertex (green dot) extends beyond the detected façade segment due to occlusion, the façade is extended along its principal direction (dashed line). The optimal corner (red dot) is reconstructed at the projection of the outline endpoint onto this extended line. (**b**) Fusion results: Comparison showing the initial roof outline (gray) and the fused outline (blue), highlighting the correction of edge deviations. (**c**) Final refinement: The red outline illustrates the final geometrically rectified model, where non-parallel edges are adjusted to strictly enforce orthogonality constraints derived from the fused façades.

Reconstructing a watertight model requires merging the roof and walls. However, TomoSAR data often present a dilemma: the façades are precise but fragmented (due to shadows/occlusion), while the roof is complete but noisy. To get the best of both worlds, we use the façade footprints as the “ground truth” for orientation. We first connect adjacent walls that are close to each other to form a continuous base. When matching the roof to the walls, we frequently encounter “short walls” where the data was cut off. Instead of warping the roof to fit the short wall, we mathematically extend the wall along its straight line until it meets the roof’s corner. This allows us to reconstruct sharp, accurate building corners even when the radar did not capture the full wall data, as demonstrated in Figure 10.

## 4. Processing Method for Roof Anomalies

**Figure 11:**
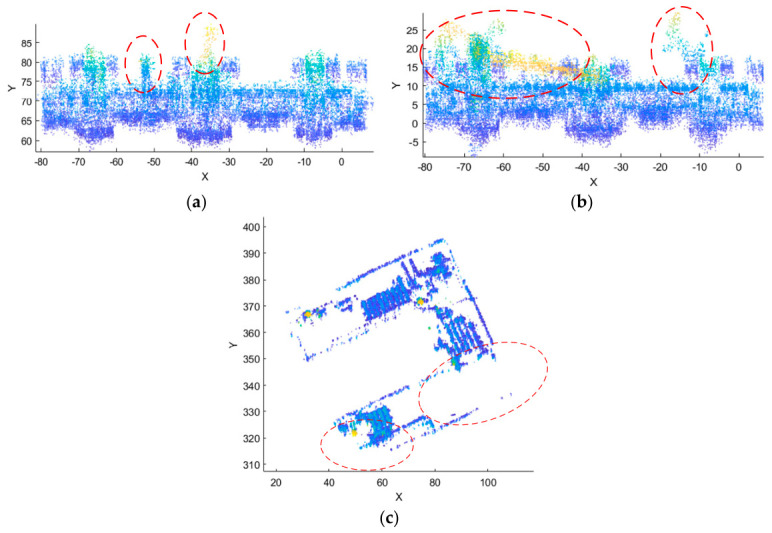
Schematic diagram of challenges in accurate roof point cloud reconstruction due to various factors: (**a**) roof anomalies caused by strong scattering; (**b**) geometric distortions resulting from interfering objects; (**c**) structural defects due to large-area data missing. The red dashed circles highlight the specific regions exhibiting these issues.

Roof point clouds frequently suffer from voids, noise, or even complete data loss [20], as illustrated in Figure 11. To address these defects, we leverage symmetry as a key geometric prior. Symmetry is a common architectural principle [43] and serves as a robust constraint to filter outliers and repair partial voids [44,45,46]. Furthermore, for extreme cases where roof data are entirely absent, we propose a parameter estimation method based on building shadow regions to enable model reconstruction.

### 4.1. Symmetrization Processing

**Figure 12:**
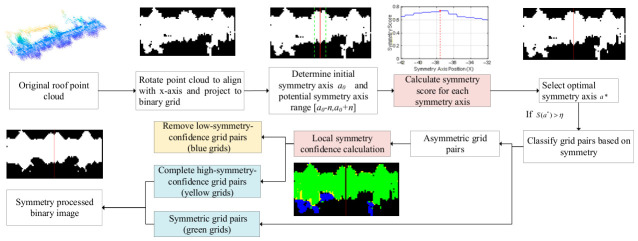
Symmetry analysis and processing workflow.

This section outlines a geometric regularization method using symmetry constraints to refine roof outlines, specifically targeting outlier removal and void completion. The process consists of three stages: symmetry axis estimation, confidence evaluation, and morphological reconstruction. The symmetry analysis and processing flow is shown in Figure 12.

First, the roof point cloud is aligned with the principal direction (*x*-axis) and projected onto the *xy*-plane to form a binary occupancy grid G(x,y), where G=1 indicates the presence of points. For the projected roof point cloud, we establish an initial symmetry axis defined by the line $x=a_{0}$, where $a_{0}$ represents the *x*-coordinate of the geometric center of the roof boundaries. Subsequently, the optimal axis position $a^{*}$ is sought within a localized range $\left[a_{0}-\delta ,a_{0}+\delta \right]$. For a candidate axis x=a, the grid is split into a left region $L_{a}$ and a mirrored right region $R_{a}$. We evaluate the structural symmetry using a score S(a), formulated as an intersection-over-union (IoU) metric:

(8) $$S(a)=\frac{\sum_{j,k}\left(L_{a}(j,k)\cdot R_{a}(-j,k)\right)}{\sum_{j,k}\max(L_{a}(j,k)\cdot R_{a}(-j,k))}$$

where $L_{a}\cdot R_{a}$ represents the overlapping (symmetric) elements, and the denominator represents the union of the two halves. The optimal axis is determined by $a^{*}=\arg\max_{a\in [a_{0}-\delta ,a_{0}+\delta ]}S(a)$. If $S(a^{*})>\eta$ (validity threshold), the roof is deemed symmetric.

For the classification of grid pairs symmetric to $a^{*}$, we define symmetric pairs ($S_{pairs}$) as locations where both mirrored sides contain data (i.e., G(j,k)=G(−j,k)=1 in relative coordinates), and asymmetric pairs ($A_{pairs}$) as locations where only one side has data.

For each asymmetric pair at location (j,k), we calculate a local symmetry confidence, C(j,k), by assessing the spatial consistency of its neighborhood. This is defined as the number of confirmed symmetric pairs within an r×r neighborhood $\Omega_{\left(j,k\right)}$:

(9) $$C(j,k)=\sum_{\left(j^{\prime },k^{\prime }\right)\in \Omega_{\left(j,k\right)}}I\left(\left(j^{\prime },k^{\prime }\right)\in S_{\mathrm{pairs}\;}\right)$$

where $I\left(\cdot \right)$ is the indicator function, and $\Omega_{\left(j,k\right)}$ denotes the local window centered at (j,k).

Based on the confidence C(j,k) and a decision threshold ζ, we execute the final regularization. In cases of high confidence (where C(j,k)≥ζ), the asymmetry is attributed to data loss (e.g., occlusion), and the missing side is filled (set to 1) to restore symmetry. Conversely, for low-confidence cases (where C(j,k)<ζ), the isolated points are attributed to noise or artifacts, and the existing points are removed (set to 0) to clean the outline.

### 4.2. Parameter Estimation Based on Building Shadow Regions

For buildings where roof point clouds are entirely absent (due to specular reflection or occlusion), we propose a physics-based inversion method. This approach exploits the geometric relationship between the building structure and its radar shadow cast on the ground to estimate the roof width W.

**Figure 13:**
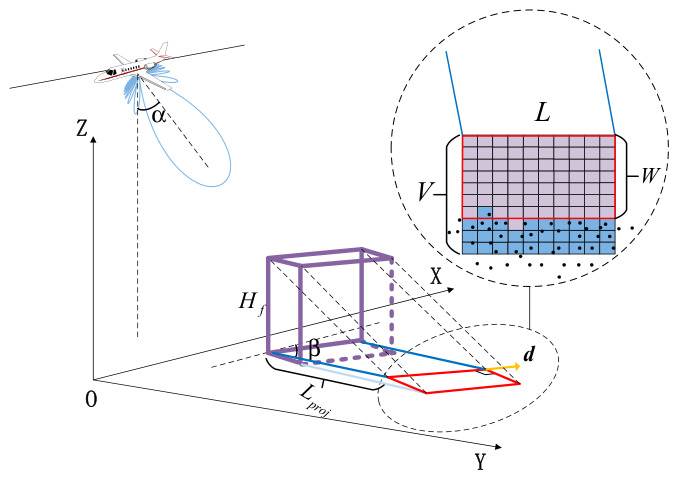
Schematic diagram of roof width estimation based on shadow analysis. Geometric definitions: $H_{f}$ is the estimated façade height; α is the radar incidence angle; $L_{proj}$ represents the projected length of the façade shadow; and d is the principal direction vector. Shadow extraction process: The blue dashed box indicates the projection of the façade shadow, which is excluded from the estimation. The red rectangular frame defines the search region (ROI) with size L×V, extended from the façade shadow boundary along the projection direction. Grid analysis: Within the ROI, purple grids represent effective void cells, while blue grids denote non-void cells (where the black dots represent the point clouds), and W is the final estimated roof width derived from S.

The estimation process is modeled as a geometric inverse problem involving three steps: façade shadow exclusion, ROI determination, and width inversion. The detailed methodology is illustrated in Figure 13.

**Step 1:** Since the ground void is a composite of shadows from both the façade and the roof, we must first isolate the component attributable to the roof. Let $H_{f}$ be the estimated height of the primary façade and α be the radar incidence angle. The theoretical ground projection length of the façade shadow, $L_{proj}$, along the radar line-of-sight (LOS) direction is calculated as

(10) $$L_{proj}=H_{f}\cdot \tan\alpha$$

By projecting the bottom baseline of the façade along the LOS direction by distance $L_{proj}$, we locate the projected façade top line, which serves as the starting boundary for roof shadow analysis.

**Step 2:** We define a search region of interest (ROI) to capture the potential roof shadow. Starting from the projected façade top line, we extend a rectangular area outward along the projection direction. The extension length is defined by a preset hyperparameter V (e.g., V=50 m), representing the maximum plausible search distance. This forms a candidate region of size L×V, where L is the length of the façade footprint.

**Step 3:** Within the ROI, we discretize the space into a grid mesh. We identify the “void” clusters (grid cells with no point cloud data) and extract the largest connected component as the effective roof shadow area, denoted as S. Under the assumption of a rectangular roof geometry, the roof width W is derived by normalizing the shadow area by the façade length $W=\frac{S}{L}$. Finally, the estimated width is validated against physical constraints $W\in [W_{\min},\;W_{\max}]$ to reject outliers.

While the proposed shadow-based inversion offers a viable solution for recovering parameters in the absence of roof data, it is subject to certain geometric and environmental limitations. First, the reliance on a rectangular roof prior—derived from typical urban residential typologies—may constrain its generalizability to non-standard architectural structures, such as those with free-form or highly irregular roof geometries. Second, the robustness of shadow region extraction is contingent upon ground point cloud density. In low-reflectivity environments (e.g., vegetation or grasslands), weak backscattering can lead to sparse ground coverage, potentially compromising the definition of shadow boundaries. However, in the target application domain of urban areas, hard-paved surfaces (e.g., concrete and asphalt) typically yield abundant backscatter. This ensures sufficient ground point density, maintaining the method’s high practicality for reconstruction in low-density rectangular building clusters. In future studies, fusing SAR intensity data—where shadows explicitly appear as dark spots—could further enhance the robustness of shadow delineation against severe multipath or mirror-like reflection interference.

## 5. Experimental Results

To demonstrate the effectiveness and urban adaptability of the proposed structure-prior-based reconstruction method, experimental validations were conducted using TomoSAR point clouds from both an individual U-shaped building and urban building clusters.

### 5.1. Experiment on an Individual Building with Complex Geometries

**Figure 14:**
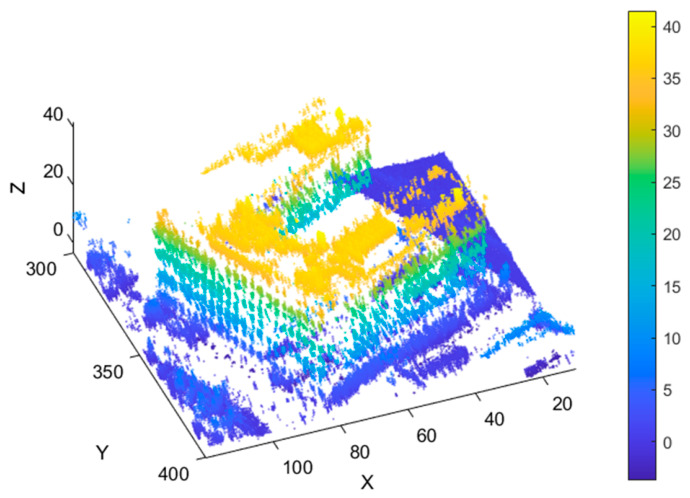
Point cloud of a building in Suzhou.

The experimental data were sourced from the publicly available SARMV3D-3.0 dataset published by the Chinese Academy of Sciences in the Journal of Radars [47]. The airborne TomoSAR point clouds were generated through tomographic synthetic aperture radar imaging of selected urban areas in Suzhou, China, from which individual building point clouds were extracted for experimentation. Figure 14 displays the representative point cloud data used in this section.

**Figure 15:**
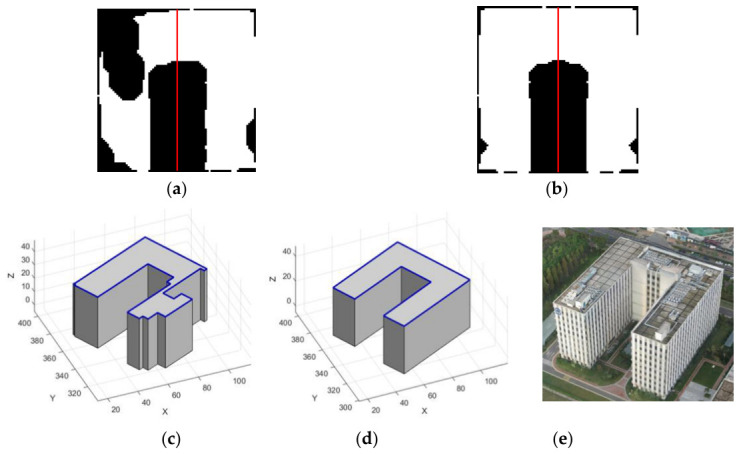
Reconstruction model results of a U-shaped building in Suzhou. (**a**,**b**) are binarization results of the original point cloud projection without and with symmetrization, respectively, and the red solid line indicates the estimated symmetry axis position. (**c**,**d**) are models reconstructed by directly extracting outlines from the point cloud without and with symmetrization, respectively. (**e**) is the corresponding oblique photography reference model.

The roof point cloud exhibits partial voids in certain areas due to the flat surface geometry and lack of strong scattering features. Direct alpha shape extraction from such incomplete point clouds yields defective building outlines (Figure 15b) that deviate from the actual structure geometry. Therefore, prior knowledge is essential to complete these void regions.

Figure 15 compares reconstruction results with/without symmetrization processing. The result without symmetrization processing (Figure 15d) demonstrates shape distortion from direct incomplete data usage, while the result with symmetrization processing (Figure 15e) achieves more reliable reconstruction. This confirms that symmetry priors effectively complete missing structures for realistic modeling.

### 5.2. Processing Results of Building Clusters

**Figure 16:**
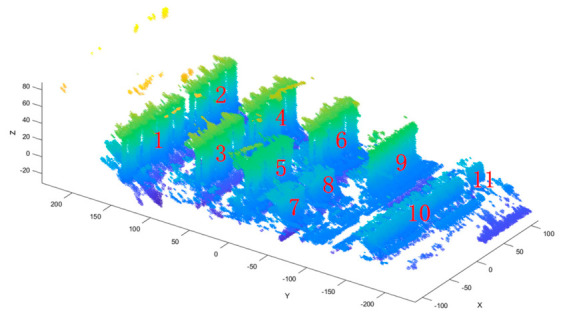
Point cloud of building clusters in Yuncheng; The red numbers indicate the IDs of individual buildings. Different colors represent different heights, and the roof point clouds of buildings 9 and 11 suffer from severe missing data.

The research data are sourced from the SARMV3D-1.0 dataset, publicly published by the Chinese Academy of Sciences in the Journal of Radar Science and Technology [48]. The airborne TomoSAR point clouds cover parts of the urban areas in Yuncheng City, Shanxi Province, and Emei City, Sichuan Province, China. Figure 16 presents the point cloud data of Yuncheng used in this section.

**Figure 17:**
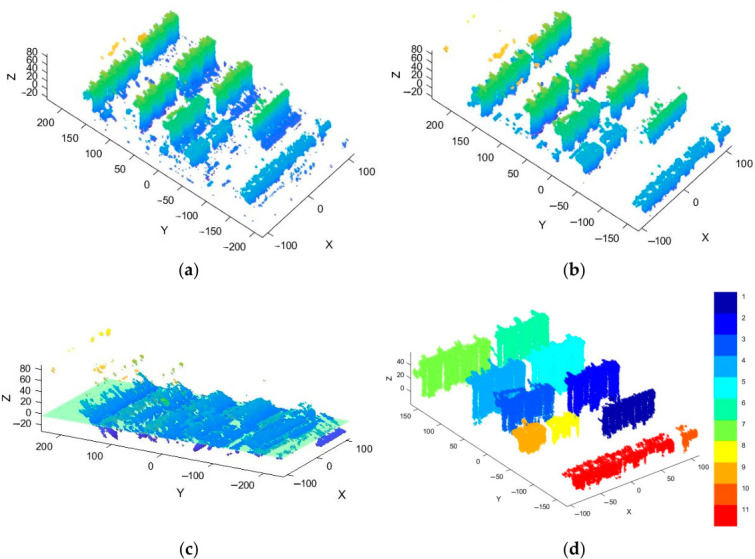
Result of building point cloud extraction: (**a**) building point cloud obtained by SMRF filtering; (**b**) building point cloud obtained by combining point cloud projection density and ground plane height; (**c**) ground plane fitting results using segmented non-building point clouds; (**d**) density clustering results. In (**a**–**c**), colors represent the elevation of the point clouds. In (**d**), different colors indicate different individual building clusters.

The point clouds were first processed using the SMRF method for preliminary segmentation of building and ground points. A plane-fitting algorithm was then applied to the ground points to estimate ground surface parameters. Figure 17a shows the filtering results of the SMRF method. Most multipath artifacts and ground points were successfully removed, although some ground points and residual multipath clusters remained near the buildings. Figure 17b presents the results after additional filtering based on the fitted ground parameters and point density, which effectively separated ground points and multipath interference from the building point clouds. The density-based spatial clustering of applications with noise (DBSCAN) algorithm was then applied to isolate individual building point clouds. Figure 17d illustrates the clustering results, with different buildings successfully separated to provide a reliable foundation for subsequent processing.

**Figure 18:**
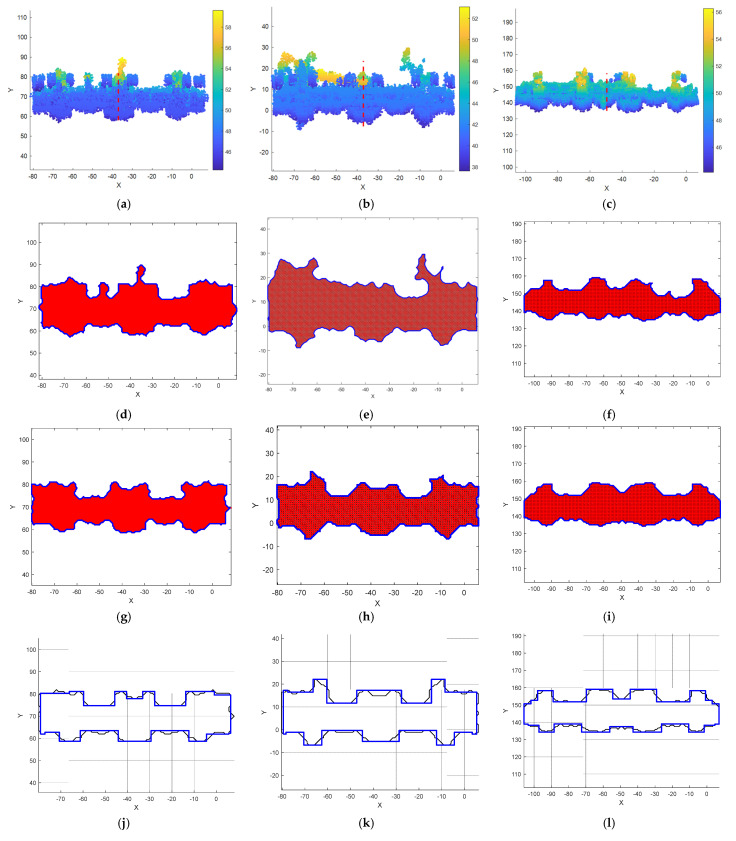

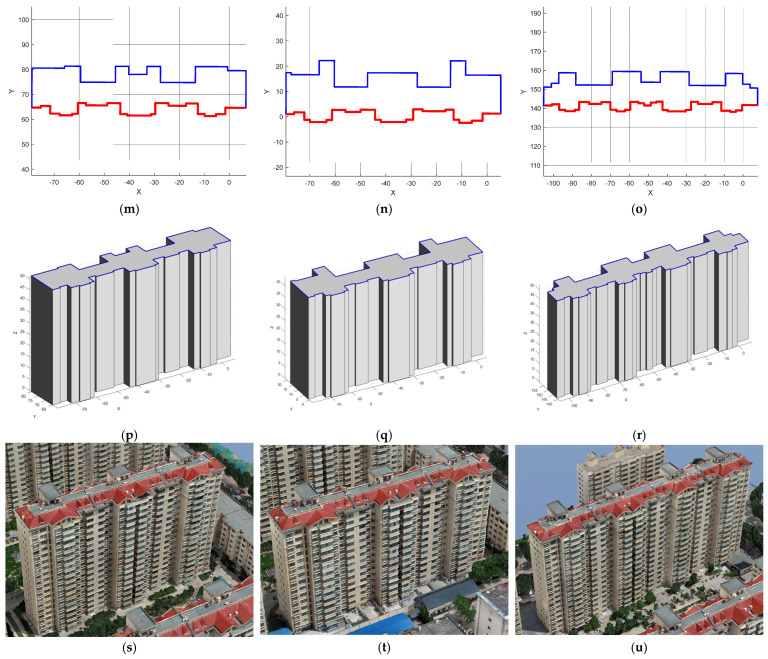
Building model extraction for Buildings 1, 3, and 5 in Yuncheng dataset. (**a**–**c**) are the point clouds before symmetrization processing, where the red dashed line indicates the estimated symmetry axis, and different colors represent point clouds at different heights. (**d**–**f**) are alpha shape extraction results directly from original point clouds. (**g**–**i**) are alpha shape extraction results after symmetrization processing. (**j**–**l**) are comparisons between roof outlines obtained by orthogonal regularization processing and alpha shapes, where black lines represent alpha shape outlines, blue lines represent orthogonalized outlines, and blue vertices represent vertices after orthogonal processing. (**m**–**o**) are outlines after façade integration, where red lines represent refined reconstructed façades, and blue lines represent roof outlines obtained by orthogonal regularization processing. (**p**–**r**) are final model reconstruction results. (**s**–**u**) are oblique photography reference models.

Figure 18 shows reconstruction results for three buildings in the Yuncheng dataset, with corresponding oblique photogrammetry reference models in Figure 18s–u. Figure 18d–f present outline extraction results from direct application of the alpha shape algorithm to projected roof point clouds. The irregular point distribution causes the extracted outlines to deviate significantly from actual building shapes. Therefore, the raw point clouds require preprocessing before outline extraction. Figure 18g–i illustrate the alpha shape results after symmetrization processing for these three buildings. Following symmetry-based refinement, the extracted outlines show markedly improved regularity and align closely with actual building geometries. This demonstrates that symmetric geometric priors enable point cloud completion and provide global constraints, effectively mitigating asymmetrical artifacts from data voids and noise, thus yielding more accurate reconstructions. Additionally, fusing refined façade information enhances the final outlines with greater detail, as shown in Figure 18m–o. Finally, incorporating estimated height parameters produces the complete 3D building models shown in Figure 18p–r.

**Figure 19:**
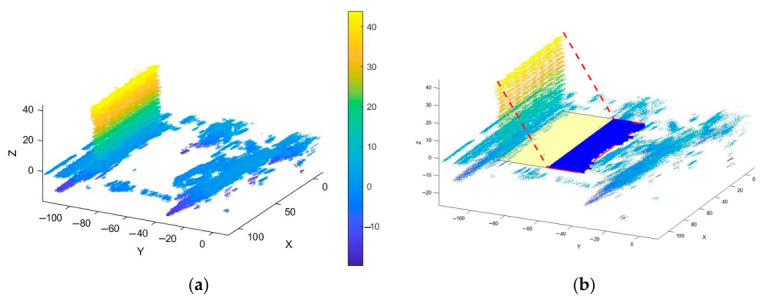
Roof width estimation without roof point cloud. (**a**) Building point cloud to be estimated, with different colors representing different heights. (**b**) Schematic diagram of roof width estimation. Yellow rectangle represents façade shadow, red dashed line represents electromagnetic wave incidence direction, blue grid represents maximum clustering result of ground blank area, and red solid line represents estimated rectangular roof.

Buildings 9 and 11 shown in Figure 16 suffer from severe roof data deficiency, rendering direct outline extraction impossible. We applied the proposed shadow-based method to reconstruct these missing parameters. Figure 19 visualizes the process: the prominent ground void adjacent to the projected façade shadow (yellow) is identified as the roof-induced shadow. By extracting the effective shadow area (the blue grid region in Figure 19b), the roof width was successfully derived to complete the model reconstruction.

To quantitatively validate this approach, we performed blind tests on Buildings 3–8, utilizing only façade points despite the availability of roof data. Table 1 compares the estimated parameters against LiDAR reference values. The results demonstrate high agreement, confirming the method’s effectiveness. Notably, Building 9 exhibits larger deviations; this is attributed to sparse façade data, which compromised the height estimation and subsequently propagated errors to the width estimation.

To evaluate the façade reconstruction quality, we compared the proposed piecewise refined fitting approach with the traditional simple planar fitting method (as typically used in [20]). While simple planar fitting provides basic edge constraints, it often relies on straight-line approximations that fail to capture fine geometric details of complex façades. In contrast, our method leverages the high density of façade point clouds to extract more reliable structural information, thereby enhancing the geometric detail of the reconstructed outlines.

Table 2 presents the quantitative comparison of these two methods using the root mean square error (RMSE) metric. Calculated as the spatial deviation between the reconstructed façade footprints and the original point cloud data, the RMSE serves as a direct indicator of geometric fidelity. The results demonstrate that our piecewise refined fitting approach yields significantly lower RMSE values compared to simple planar fitting across most test buildings. This indicates that the proposed method achieves superior conformity to the actual point cloud distribution, particularly for buildings with non-flat façade structures.

**Figure 20:**
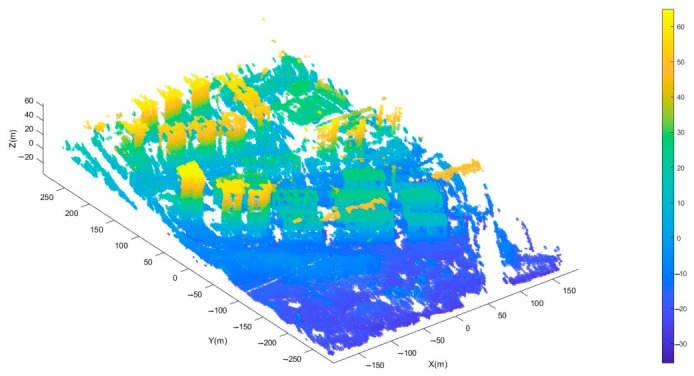
Overview of the airborne TomoSAR point cloud for the building clusters in the Emei dataset. Colors represent elevation.

To further validate the generalization capability of the proposed method, we conducted experiments on another scene covering the urban area of Emei City in the SARMV3D-1.0 dataset. The corresponding TomoSAR point cloud is illustrated in Figure 20, which depicts a dense cluster of residential buildings with varying heights and complex spatial arrangements. In this challenging scenario, we comprehensively evaluated the reconstruction performance by comparing the proposed method against two representative state-of-the-art approaches: the data-driven alpha shape method in [20] and the geometric-primitive-based method in [49].

**Figure 21:**
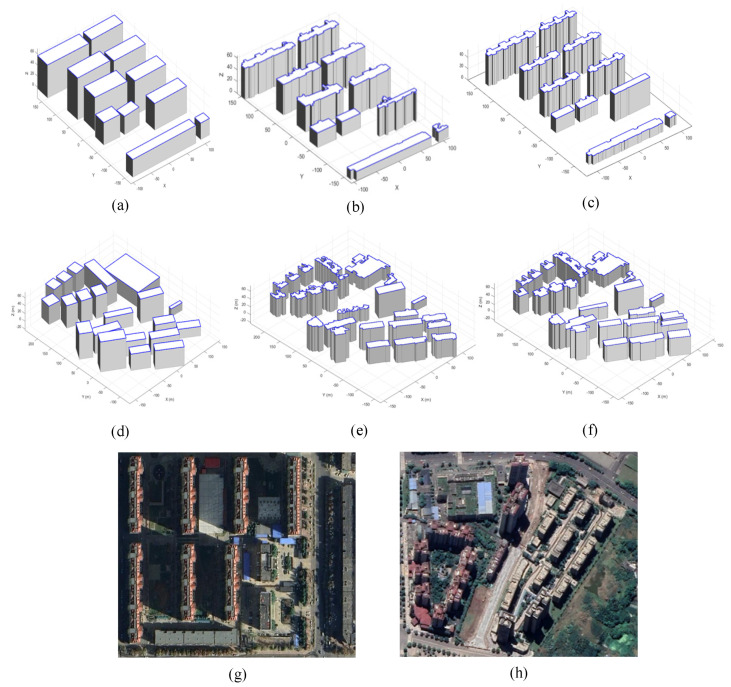
Visual comparison of 3D building reconstruction results on Yuncheng (**top row**) and Emei (**bottom row**) datasets. Columns from left to right display: (**a**,**d**) results of the region-based box model method in [49]; (**b**,**e**) results of the alpha-shape-based method in [20]; (**c**,**f**) results of the proposed method; and (**g**,**h**) reference optical images. The blue lines indicate the reconstructed roof outlines.

Figure 21 presents the reconstruction results of these three methods. As shown in the first column (Figure 21a,e), the method in [49] adopts a strict box-like assumption. While this ensures high regularity, it tends to oversimplify complex building footprints, failing to capture fine-grained variations along the façades (e.g., the U-shaped or stepped structures in the Yuncheng dataset). Conversely, the method in [20] (Figure 21b,f) relies heavily on point density. Without sufficient regularization, it produces jagged and irregular boundaries, making the models visually noisy.

In contrast, the proposed method (Figure 21c,g) effectively overcomes these limitations. By integrating piecewise refined fitting with orthogonality and symmetry priors, our approach preserves intricate architectural details (superior to [49]) while maintaining clean, sharp outlines (superior to [20]). The results are highly consistent with the optical references (Figure 21d,h), demonstrating the robustness of our method in complex urban scenarios.

To quantitatively evaluate the efficiency, we recorded the total runtime of the proposed method and the comparative approaches [20,49] on the Yuncheng and Emei datasets. All experiments were conducted on a mobile workstation equipped with an 11th Gen Intel i9-11950H CPU and an NVIDIA RTX A4000 GPU. The proposed method and the baseline algorithms [20,49] were implemented in MATLAB R2021a and executed under the same computing environment to ensure a fair comparison. As shown in Table 3, the proposed framework demonstrates competitive performance.

As observed, compared to the graph-cut-based method [20], our approach reduces the processing time by approximately 30–40%. This significant speedup is attributed to the adoption of deterministic geometric operations (i.e., SMRF filtering and shadow projection), which avoid the computationally expensive global energy minimization and iterative optimization inherent in [20]. It is worth noting that while Wang et al. [49] achieve faster runtimes, their efficiency largely relies on a sparse sampling strategy and 2D-domain segmentation. In contrast, our method processes the full-resolution 3D point cloud without downsampling the input data during the geometry recovery stage. Although this incurs a marginal time cost, it ensures that the reconstruction is driven by dense structural observations, thereby offering superior robustness and detail preservation as demonstrated in the qualitative comparisons. Overall, the proposed method strikes an optimal balance between computational speed and reconstruction quality.

### 5.3. Ablation Study

**Figure 22:**
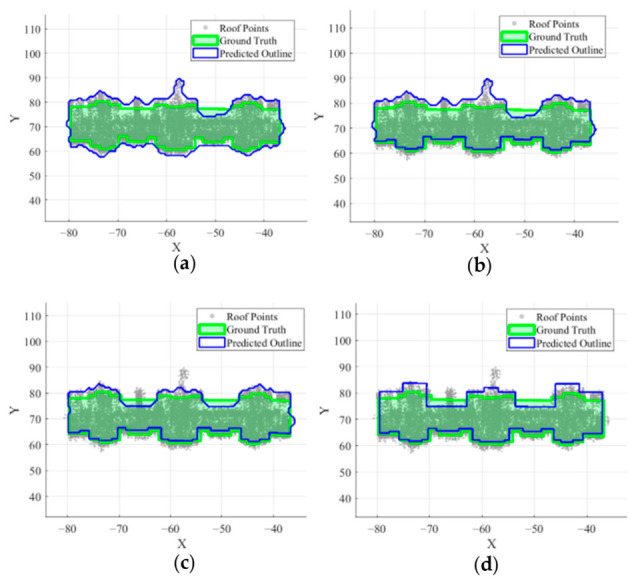
Visual evolution of the reconstructed outline (Building 3) during the ablation study. Gray dots: input roof point cloud; green line: registered ground truth; blue line: predicted outline. (**a**) Baseline alpha shape extraction. (**b**) Outline with façade regularization. (**c**) Outline after symmetry processing. (**d**) Final outline generated by the full proposed framework.

To evaluate the contribution of each geometric prior, we conducted an ablation study on a complex structure (Building 3 in the Yuncheng dataset). This building features a stepped U-shaped boundary with severe side-lobe artifacts from strong scatterers, providing an effective case to analyze the rectification of topological defects. Four configurations were evaluated: (a) baseline alpha shape extraction, (b) addition of façade regularization, (c) addition of symmetry processing, and (d) the full proposed framework. Quantitative metrics (IoU and vertex count) and the corresponding visual results are presented in Table 4 and Figure 22, respectively.

As illustrated in Figure 22, the baseline alpha shape method (Figure 22a) directly delineates the raw roof points. Affected by multipath scattering and side-lobe artifacts, it generates a highly irregular polygon containing a massive noise cluster at the top edge. Adding façade footprints (Figure 22b) straightens the boundaries aligned with the observed walls, yet the top side-lobe artifacts remain distorted. The introduction of the symmetry prior (Figure 22c) successfully identifies and removes this asymmetric noise cluster, restoring the global U-shaped balance. Finally, orthogonal regularization (Figure 22d) enforces architectural regularity, rectifying rounded corners to match the true rectilinear topology.

The quantitative results in Table 4 further validate the contribution of each module. The baseline method yields an IoU of 0.65 with 283 vertices, as its bloated shape merely envelopes the dilated radar scatterers and noise without architectural constraints. By progressively introducing façade, symmetry, and orthogonal priors, the IoU steadily improves to 0.78. More importantly, the vertex count decreases significantly from 283 to a minimal set of 54. This massive reduction in vertices, combined with the continuous IoU improvement, demonstrates that the proposed framework not only increases the area overlap but effectively eliminates hundreds of jagged edges to reconstruct a topologically correct architectural model.

### 5.4. Qualitative Comparison with GlobalBuildingAtlas

**Figure 23:**
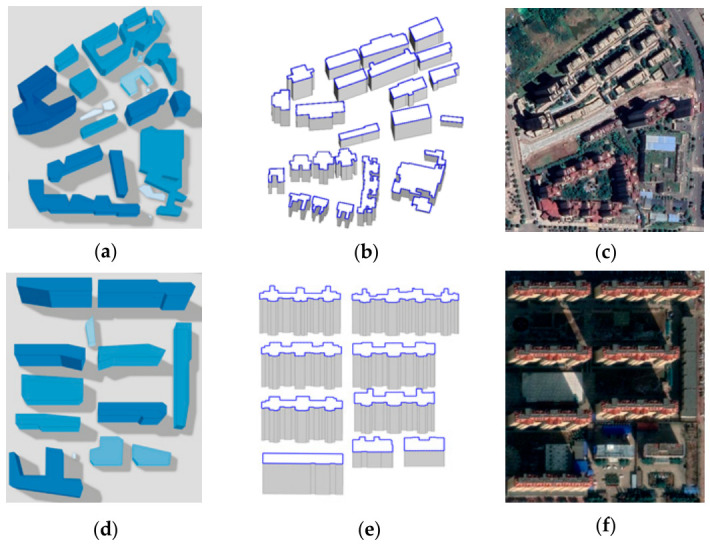
Qualitative comparison of building reconstruction in the Emei (**top row**) and Yuncheng (**bottom row**) study areas. (**a**,**d**) Standard LoD1 3D building models provided by GlobalBuildingAtlas, showing generalized bounding polygons. (**b**,**e**) Refined 3D models reconstructed by the proposed method, capturing detailed topological structures. (**c**,**f**) Corresponding optical satellite imagery serving as morphological references. In (**a**,**d**), the blue polygons represent the building models from GlobalBuildingAtlas. In (**b**,**e**), the blue lines indicate the reconstructed roof outlines.

To evaluate the reconstruction detail of the proposed framework, we present a qualitative comparison with GlobalBuildingAtlas [50], an open-source global dataset of building polygons and Level of Detail 1 (LoD1) 3D models. Figure 23 illustrates this comparison using urban patches from both the Emei and Yuncheng datasets.

As shown in Figure 23a,d, GlobalBuildingAtlas effectively captures macro-level urban layouts using simplified bounding polygons typical of standard LoD1 models. However, due to its global processing scale, it inherently tends to oversimplify complex architectural footprints. It frequently merges adjacent structures and omits fine geometric variations, such as the U-shaped indentations in the Emei area (Figure 23a) and the repeating stepped facades in the Yuncheng area (Figure 23d).

In contrast, Figure 23b,e demonstrate that the proposed TomoSAR-based method recovers fine-grained structural details that align closely with the true building morphology presented in the corresponding optical imagery (Figure 23c,f). This comparison illustrates a practical complementary relationship: large-scale datasets like GlobalBuildingAtlas provide extensive LoD1 baselines, while localized algorithms can further refine specific complex urban patches, effectively upgrading generalized footprint representations to higher geometric fidelity.

## 6. Discussion

### 6.1. Discussion on Parameter Selection

Although the proposed framework involves several parameters to handle the complexity of TomoSAR point clouds, most of them are not arbitrary empirical values but are strictly related to the physical attributes of the scene (e.g., spatial resolution, building scale) or are determined adaptively. Following the categorization in [20], we discuss the selection of key parameters and their impact on reconstruction stability.

Density and Depth Jump Thresholds ($\rho_{th}$ and μ): To separate true façade points from multipath noise, the density threshold $\rho_{th}$ is determined dynamically based on the average grid density $\rho_{avg}$ of the projected ground plane (e.g., $\rho_{th}=3\rho_{avg}$). For the piecewise façade fitting, the depth jump threshold μ is set based on typical architectural variations (e.g., balconies or setbacks) and the radar range resolution. Variations smaller than μ (set to 1.0 m in our study) are treated as planar noise.

Effective Roof Width Threshold ($W_{eff\_th}$): To trigger the shadow-based parameter estimation for extreme cases, we evaluate the effective roof width, calculated as the extracted roof area divided by the façade length ($W_{eff}=S_{extracted}/L$). Since standard urban buildings are rarely narrower than 3 m, if $W_{eff}<3$ m, the roof data is considered severely defective, and the shadow analysis is activated.

Weighting Coefficients ($w_{p},w_{r}$): In the adaptive threshold optimization (Equation (4)), these coefficients balance geometric fidelity ($w_{p}$) against the vertex reduction rate ($w_{r}$). Unlike LiDAR data, which capture sharp building boundaries, TomoSAR building edges are often irregular due to noise and signal processing artifacts. Therefore, we recommend prioritizing model simplification over strict adherence to the noisy original boundary. In our study, assigning a higher weight to simplification (e.g., $w_{p}$ = 0.3, $w_{r}$ = 0.7) effectively filtered out high-frequency noise, yielding regularized outlines that better represent the actual architectural shapes.

Regularization Threshold ψ: The Douglas–Peucker threshold ψ governs the trade-off between outline simplification and geometric fidelity. Unlike traditional methods that fix this value empirically, our approach treats ψ as an adaptive parameter. As described in Equation (4), ψ is automatically optimized for each building by maximizing the evaluation metric M, which balances the vertex reduction rate and shape similarity. This adaptive mechanism significantly reduces the algorithm’s reliance on manual tuning and ensures generalization across buildings with varying complexities.

Symmetry Constraints (η and ζ): To prevent the generation of misleading artifacts in irregular structures, the symmetry thresholds η (symmetry score) and ζ (confidence) are set conservatively. We employ a high threshold strategy (e.g., η > 0.7) to ensure that the symmetry prior is triggered only when strong structural evidence exists. Sensitivity analysis shows that within a stable range ($\eta \in \left[0.65,\;0.8\right]$), the reconstruction results remain consistent, effectively repairing data voids while preserving true asymmetries in complex buildings.

### 6.2. Discussion on Applicability and Robustness

The proposed method leverages structural priors to mitigate severe TomoSAR noise and data voids. While orthogonality constraints optimize the approach for typical urban environments, the piecewise refined fitting strategy (Section 3.2) extends its applicability beyond simple rectangles to complex non-convex geometries (e.g., U-shaped buildings) by connecting local linear segments. For irregular or non-rectilinear structures, the algorithm adaptively relaxes these regularization constraints, shifting the focus from strict orthogonality to data fidelity. This prevents the over-simplification of unique architectural shapes into generic forms, enabling the method to process diverse urban morphologies ranging from planned grids to free-form architectural styles.

Regarding symmetry, an adaptive application strategy is employed to prevent the generation of artifacts. As detailed in Section 4.1, symmetry constraints are activated only when the data yield high confidence scores. This selective mechanism effectively repairs occlusion-induced gaps in symmetric structures while strictly preserving the observed data for inherently asymmetric buildings.

## 7. Conclusions

This paper proposes a method for urban building modeling extraction from SAR tomography point clouds by exploiting the structural prior information, allowing automated reconstruction of detailed 3D building models from TomoSAR point cloud data to be realized. The method leverages geometric prior constraints such as orthogonality and possible symmetry of buildings, effectively improving the reconstruction completeness and accuracy of building façade footprints and roof outlines. The proposed approach can also reasonably estimate building parameters, particularly in cases of incomplete point cloud data or missing information. Nevertheless, the manually summarized geometric prior knowledge in this study may not fully cover all building types. Future work will explore integrating deep learning and big data technologies to expand the applicability of geometric priors, aiming to generate 3D models that better align with real-world building structures.

## Figures and Tables

**Table 1:** Building parameter estimation results of the proposed method.

Building ID	Reference Height	Reconstructed Height	Reference Width	Width Estimated Based on Shadow Area	Height Reconstruction Error	Roof Width Estimation Error
1	54	52.7	—	—	1.3	—
2	54	53.9	—	—	1.1	—
3 *	53	51.6	19.0	21.7	1.4	2.7
4 *	53	52.0	19.0	22.7	1.0	3.7
5 *	45	43.9	18.0	16.4	1.1	1.4
6 *	51	50.8	18.0	17.7	0.2	0.3
7 *	24	24.9	14.6	13.7	0.9	0.9
8 *	21	24.4	14.4	15.4	3.4	1.0
9 *	53	48.1	14.0	17.7	4.9	3.7
10	—	15.5	—	—	—	—
11 *	—	17.0	—	11.7	—	—

* Buildings with asterisk indicate roof parameters estimated using shadow area. — Dash indicates building parameters not obtained.

**Table 2:** Comparison of RMSE values between plane and proposed polyline fitting models.

Building ID	RMSE of Plane Fitting Model	RMSE of Proposed Method Fitting Model
1	1.2987	**1.2255**
2	3.1266	**1.3952**
3	3.8406	**1.4211**
4	3.5189	**1.3553**
5	3.4929	**1.8392**
6	3.6144	**1.1016**
7	3.2011	**1.1339**
8	1.3036	**1.1947**
9	1.7592	**1.7198**
10	1.2989	**1.0640**
11	1.1829	**0.9876**

Bold values indicate the best results (lowest RMSE).

**Table 3:** Running time of different methods (seconds).

Method	Key Strategy	Yuncheng	Emei
Reference [20]	Global graph cuts optimization	86.0	126.0
Reference [49]	Sparse sampling and 2D cuts	32.0	52.0
Proposed Method	SMRF and geometric projection	50.0	72.0

**Table 4:** Quantitative results of the ablation study on Building 3.

Configuration	IoU	Vertex Count
Baseline (Alpha Shape Only)	0.65	283
+Façade Regularization	0.70	114
+Symmetry Completion	0.75	98
**Ours (Full Framework)**	**0.78**	**54**

Bold values indicate the best results.

## Data Availability

Publicly available datasets were analyzed in this study. These data can be found here: https://www.radars.ac.cn/web/data/getData?newsColumnId=9ac203a4-90ca-4e8e-8663-6b6e89cfacea&pageType=en (accessed on 26 March 2026).
